# Retraction Note: Thyroid nodules update in diagnosis and management

**DOI:** 10.1186/s40842-016-0025-9

**Published:** 2016-04-14

**Authors:** Shrikant Tamhane, Hossein Gharib

**Affiliations:** 1 Endocrinology and Metabolism, Rochester, 55905 MN USA; 2 Mayo Clinic College of Medicine, Rochester, 55905 MN USA; 3 Division of Endocrinology, Diabetes, Metabolism, and Nutrition, Mayo Clinic, 200 First Street SW, Rochester, 55905 MN USA

The authors are retracting this article [1] due to overlap with previous publications, most notably [2–5], without attribution. The authors apologise for any inconvenience.
